# 在线固相萃取-超高效液相色谱-串联质谱法快速测定地表水中5种解热镇痛药物

**DOI:** 10.3724/SP.J.1123.2024.10018

**Published:** 2025-09-08

**Authors:** Ping HE, Liqun WANG, Shan ZHOU, Baofeng ZHANG, Xuan JIA, Yi CHI, Zhenqi XU, Wei TANG

**Affiliations:** 1.浙江省杭州生态环境监测中心，浙江 杭州 310007; 1. Zhejiang Hangzhou Ecological and Environmental Monitoring Center，Hangzhou 310007，China; 2.杭州市生态环境科学研究院 （杭州市城区生态环境监测站），浙江 杭州 310014; 2. Hangzhou Institute of Ecological Environment Science （Hangzhou Urban Ecological Environment Monitoring Station），Hangzhou 310014，China

**Keywords:** 在线固相萃取, 超高效液相色谱-串联质谱, 解热镇痛药物, 地表水, online solid phase extraction （online SPE）, ultra-high performance liquid chromatography-tandem mass spectrometry （UHPLC-MS/MS）, antipyretic analgesics, surface water

## Abstract

布洛芬等解热镇痛药物在日常生活中被广泛应用，其残留物可通过多种途径进入环境，对人类健康和生态环境具有潜在威胁。基于在线固相萃取-超高效液相色谱-串联质谱技术（online SPE-UHPLC-MS/MS），建立了一种快速筛查和检测地表水中布洛芬、氨基比林、安替比林、非那西丁和萘普生等5种常用解热镇痛药物的方法。样品经0.2 µm再生纤维素滤膜过滤，移取5 mL并加入一定量的内标和Na_2_EDTA，混匀，使用在线固相萃取系统自动上样，PLRP-S在线固相萃取柱富集净化。利用ZORBAX Eclipse Plus C18色谱柱（100 mm×3.0 mm，1.8 µm）进行分离，以甲醇-乙腈（1∶1，v/v）和0.2 mmol/L氟化铵水溶液为流动相进行梯度洗脱，采用电喷雾离子源，正、负离子扫描模式和多反应监测（MRM）模式检测，内标法定量。结果显示，采用该方法，5种解热镇痛药物在各自的线性范围内线性关系良好（*r*>0.998），方法检出限（MDL）为0.05~0.20 ng/L，方法定量限（MQL）为0.20~0.80 ng/L，在低、中、高3个加标水平下的回收率为64.2%~112%，相对标准偏差为2.06%~8.99%。采用该方法对取自钱塘江杭州段的6份水样进行检测，除氨基比林外，其余4种解热镇痛药物均有检出。该方法实现了大体积进样、在线富集净化及定量分析，简化了前处理步骤，15 min即可完成一个样品的测定，显著提高了检测效率，具有低检出限、高灵敏度、快速分析和操作简便的特点，适用于地表水中5种解热镇痛药物的快速测定。

## Abstract (en)

Antipyretic analgesics are typical pharmaceutical and personal care products （PPCPs） that are widely used in our daily life because they relieve fever and pain， and have anti-inflammatory and anti-rheumatic properties. These drugs inhibit the synthesis and release of prostaglandins （PGs） in the neurons of the anterior hypothalamus and exert therapeutic effects as a consequence. However， these drugs are relatively commonly misused and abused， often owing to a lack of proper medication guidance. As a result， these drugs enter the environment via various pathways， including wastewater treatment plants， agricultural runoff， and improper disposal， thereby posing potential threats to human health and ecosystems. The presence of these contaminants in surface water has become an environmental safety concern that necessitates the development of rapid， accurate， and high-throughput analysis methods. In this study， an analytical method was established for the determination of five antipyretic analgesics （ibuprofen， aminophenazone， antipyrine， phenacetin， and naproxen）. The developed method is based on online solid phase extraction coupled with ultra-high performance liquid chromatography-tandem mass spectrometry （online SPE-UHPLC-MS/MS）， which provides a high degree of automation and efficiency. Water samples were collected and filtered through 0.2-μm regenerated cellulose （RC） filter membranes， after which Na_2_EDTA and an internal standard were added. An aliquot （0.9 mL） of each sample was injected into the online SPE system using an automatic sampler. Samples were first adsorbed on a PLRP-S online SPE column， washed with 0.05% formic acid aqueous solution， and finally gradient-eluted with a mobile phase composed of 0.2 mmol/L ammonium fluoride solution and methanol-acetonitrile （1∶1， v/v）. Analytes were separated on a ZORBAX Eclipse Plus C18 column， detected by multiple reaction monitoring with electrospray ionization in both positive- and negative-ion modes， and quantified using the internal-standard method.The five antipyretic analgesics were effectively separated under the optimized experimental conditions and showed good linearities within their respective concentration ranges， with correlation coefficients （*r*） greater than 0.998. The method detection limits （MDLs） ranged from 0.05 to 0.20 ng/L， and the method quantification limits （MQLs） ranged from 0.20 to 0.80 ng/L. The five antipyretic analgesics exhibited average recoveries of between 64.2% and 112%， with relative standard deviations （RSDs， *n*=6） of 2.06%-8.99% at low， medium， and high spiked levels. Furthermore， the method was successfully used to analyze water samples from the Hangzhou section of the Qiantang River， in which four target compounds were detected， with antipyrine found to have the highest mass concentration. This newly developed method features a high degree of automation， facilitates the injection of large volumes， and enables online enrichment， purification， and quantitative analysis in an integrated process， with minimal operational errors， high repeatability， and high sensitivity. The developed method shortens sample-analysis times， is more cost-effective， and meets the needs of large-scale sample testing， as it requires only 15 min to completely determine a sample， thereby significantly improving detection efficiency. Overall， the method features low detection limits， analyzes rapidly， and is easy to operate； hence it is suitable for rapid risk screening and the quantitative determination of antipyretic analgesics in surface water.

近年来，药品和个人护理品（pharmaceutical and personal care products，PPCPs）由于其潜在的环境风险和在水环境中的普遍检出引起了广泛关注^［1-3］^。解热镇痛药（antipyretic analgesics）作为一类典型的PPCPs，在临床上广泛应用，也称为非甾体抗炎药（NSAIDs），它们通过抑制下丘脑前部神经元中前列腺素（PGs）的合成和释放，发挥解热和镇痛的双重功效，此外还有显著的抗炎、抗风湿等作用^［4］^。我国是生产和消费解热镇痛药的大国，不仅临床上普遍应用，也是各大药房销量较大的药品。然而，由于缺乏正确的用药指导，这些药物的误用和滥用现象较为普遍^［5］^。未被人体吸收、代谢或使用的解热镇痛药物，通过多种途径进入地表水，对人类健康和生态系统构成了潜在威胁^［6，7］^。

目前，国内外水中解热镇痛药物的检测方法主要基于高效液相色谱（HPLC）或液相色谱-串联质谱（LC-MS）技术^［8-12］^。这些方法的前处理技术包括液液萃取、固相萃取和固相微萃取等，通常以离线方式进行，这些传统方法耗时长，操作复杂，并且消耗大量的溶剂和耗材。近年来，在线固相萃取技术（online SPE）兴起，与LC-MS的联用也逐渐得到应用^［13-16］^。Online SPE技术具有高度自动化的特点，实现了大体积进样、在线富集净化及定量分析一体化，减少了实验操作误差，提高了重复性和灵敏度，缩短了样品分析时间，降低了实验的经济成本，能够满足大规模样品检测的需求。

本文基于在线固相萃取-超高效液相色谱-串联质谱技术（online SPE-UHPLC-MS/MS），建立了一种同时检测地表水中布洛芬、氨基比林、安替比林、非那西丁、萘普生等5种解热镇痛药物的快速定量方法，从样品上样至检测完成仅需15 min，可用于地表水中解热镇痛药物的快速风险筛查和日常监管。

## 1 实验部分

### 1.1 仪器、试剂与材料

1290 Infinity Ⅱ-G6470A超高效液相色谱-三重四极杆质谱仪，配全自动在线固相萃取系统（美国Agilent公司）；Milli-Q超纯水机（美国Millipore公司）；KQ-600DB型数控超声波清洗器（昆山市超声仪器有限公司）；0.2 µm再生纤维素滤膜（RC，美国Agilent公司）。

实验用水为超纯水（电阻率≥18.2 MΩ·cm，Milli-Q超纯水器制得）；甲醇、乙腈、甲酸（质谱纯）和乙二胺四乙酸二钠（Na_2_EDTA，色谱纯）均购自北京百灵威科技有限公司；氟化铵（分析纯，纯度98%，上海麦克林生化科技有限公司）；乙酸铵（色谱纯，纯度99.0%，上海阿拉丁生化科技股份有限公司）。

5种解热镇痛药混合标准品（含布洛芬、氨基比林、安替比林、非那西丁和萘普生，溶剂为甲醇，质量浓度为100 µg/mL）购自美国A Chemtek公司；内标物（布洛芬-D_3_，纯度99.9%）购自上海安谱璀世标准技术服务有限公司。

购买的标准溶液直接使用；使用时用甲醇稀释配制成中间使用液；系列标准工作溶液则用纯水配制。内标标准品用甲醇配制成100 mg/L的内标标准储备液，使用时用甲醇稀释至所需浓度。所有标准溶液和储备液均避光保存于-20 ℃环境下。

### 1.2 样品前处理

水样摇匀，经 0.2 µm再生纤维素滤膜过滤，去除悬浮颗粒，准确移取5 mL滤液至6 mL进样瓶中，加入10 µL内标溶液（10.0 µg/L）和5 µL Na_2_EDTA溶液（0.1 mol/L），混匀，置于在线固相萃取系统中，待上样。

### 1.3 仪器条件

#### 1.3.1 在线固相萃取条件

PLRP-S在线固相萃取小柱（12.5 mm×2.1 mm，15~20 µm，配专用卡套，美国Agilent公司）；流动相A为0.05%甲酸水溶液；流动相B为甲醇-乙腈（1∶1，v/v）；进样量：0.9 mL。在线固相萃取梯度洗脱程序见表1，其中4.00~5.00 min为阀切换时间。

**表 1:** 在线固相萃取梯度洗脱条件

Time/min	Flow rate/（mL/min）	*φ*（Ａ）/%	*φ*（B）/%
0	0.40	100	0
0.25	1.00	100	0
4.00	1.00	100	0
5.00	1.00	0	100
10.00	1.00	0	100
10.01	1.00	100	0
13.00	1.00	100	0
15.00	0.40	100	0

A： 0.05% （v/v） formic acid aqueous solution； B： methanol-acetonitrile （1∶1， v/v）.

#### 1.3.2 色谱条件

色谱柱：ZORBAX Eclipse Plus C18色谱柱（100 mm×3.0 mm， 1.8 µm，美国Agilent公司）；流动相A为0.2 mmol/L氟化铵水溶液；流动相B为甲醇-乙腈（1∶1，v/v）；流速0.3 mL/min；柱温40 ℃。梯度洗脱程序：0~4.0 min，10%B；4.0~7.0 min，10%B~55%B；7.0~12.0 min，55%B~95%B；12.0~14.0 min，95%B；14.0~14.1 min，95%B~10%B；14.1~15.0 min，10%B。

#### 1.3.3 质谱条件

离子源：电喷雾离子源（ESI）；扫描方式：正离子模式和负离子模式；检测模式：多反应监测（MRM）模式；雾化器压力：275.8 kPa；干燥气温度：300 ℃；干燥气流量：8 L/min；鞘气温度：375 ℃；鞘气流量：11 L/min；毛细管电压：正离子模式4.0 kV，负离子模式3.0 kV；喷嘴电压：正离子模式0.5 kV，负离子模式1.5 kV。各化合物的质谱参数见表2。

**表 2:** 目标化合物的保留时间与质谱参数

Compound	Retention time/min	Precursor ion （*m/z*）	Product ions （*m/z*）	Fragmentor/V	Collision energies/eV	Ionization mode
Ibuprofen （IBU）	12.181	205.0	161.1^*^	83	5， 5	ESI^-^
Atipyrine （ANT）	7.822	189.0	77.0^*^， 147.0	90	30， 20	ESI^+^
Aminophenazone （APZ）	8.189	232.0	113.0^*^， 111.0	100	12， 10	ESI^+^
Phenacetin （PCT）	8.797	180.1	65.1^*^， 110.0	118	55， 40	ESI^-^
Naproxen （NPX）	10.729	229.0	169.0^*^， 185.0	23	36， 2	ESI^-^
Ibuprofen-D_3_ （IBU-D_3_）	12.166	208.0	164.1^*^	85	5， 5	ESI^-^

* Quantitative ion.

## 2 结果与讨论

### 2.1 质谱条件优化

根据5种解热镇痛药物的化学电离性质和结构，采用电喷雾离子源正、负离子扫描模式同时采集，将5种目标化合物及其内标的混合标准溶液（200 μg/L）注入离子源，全扫描分析确定5种化合物的母离子，再进行子离子扫描，选取丰度较高且干扰较小的2个子离子分别作为定量和定性离子，以信号最强的子离子作为定量离子，然后逐一优化各离子对的破裂电压和碰撞能量，最终确定各目标化合物的质谱参数（见表2）。

### 2.2 色谱条件优化

#### 2.2.1 色谱柱的选择

鉴于目标化合物理化性质不同且极性差异较大，故选择通用型反相色谱柱进行色谱分离。实验比较了Poroshell HPH-C18（100 mm×3.0 mm，2.7 µm）、Eclipse Plus C18（100 mm×3.0 mm，1.8 µm）和Poroshell 120 EC-C18（150 mm×3.0 mm，2.7 µm）3款色谱柱的分离效果（见图1）。结果显示，Eclipse Plus C18和Poroshell 120 EC-C18柱均能有效分离5种目标化合物，且响应信号强度基本相近。综合考虑目标化合物的保留时间和氨基比林的峰形，最终选择Eclipse Plus C18色谱柱。

**图1:**
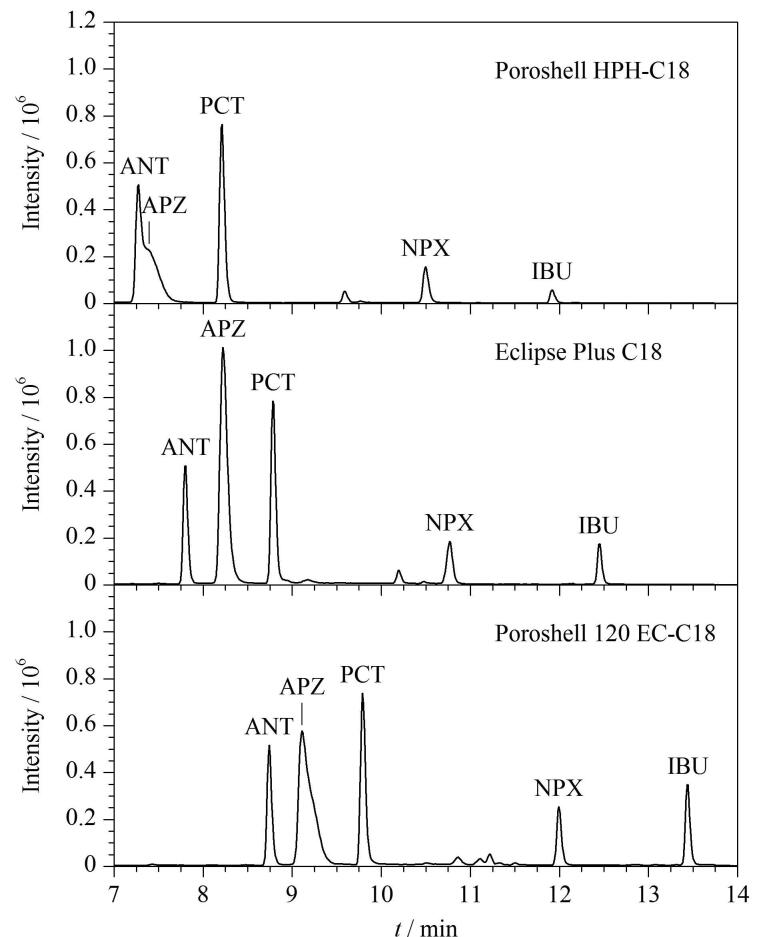
采用不同色谱柱时目标化合物的色谱图

#### 2.2.2 色谱流动相的优化

流动相的组成会影响目标化合物的色谱峰形、分离效果及离子化效率。甲醇和乙腈是色谱分析中最常用的有机流动相，甲醇作为质子性溶剂，可增强化合物的质谱响应；乙腈黏度较低，有助于降低柱压且洗脱能力较强^［17］^。实验发现使用甲醇-乙腈（1∶1，v/v）作为有机相可以获得更好的分离效果和峰形，因此有机相选择甲醇-乙腈（1∶1，v/v）。在流动相中加入适量缓冲盐可提高目标化合物的离子化效率，增强响应信号。以甲醇-乙腈（1∶1，v/v）为有机相，比较了0.5 mmol/L乙酸铵水溶液、0.2 mmol/L氟化铵水溶液和纯水这3种水相流动相（如图2所示），水相中添加一定量的氟化铵，目标化合物分离效果好，响应高，峰形对称。最终确定液相色谱的流动相为甲醇-乙腈（1∶1，v/v）和0.2 mmol/L氟化铵水溶液。

**图2:**
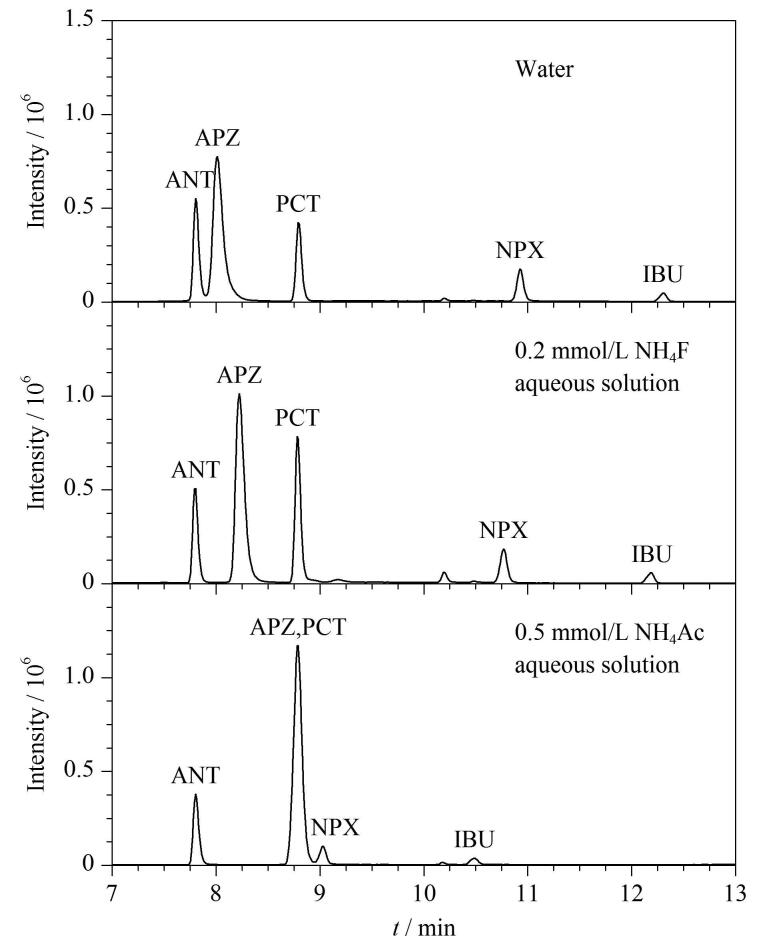
目标化合物在不同水相体系下的色谱图 Organic phase： methanol-acetonitrile （1∶1， v/v）.

### 2.3 前处理条件优化

#### 2.3.1 微孔滤膜的选择

水样的洁净度对在线固相萃取柱的使用寿命影响较大，而且样品中的微小颗粒也会造成仪器管路的堵塞，需预先使用微孔滤膜对水样进行过滤处理。由于不同材质的微孔滤膜对各目标分析物的吸附效应不同，本实验分别考察了市面上常见的尼龙（Nylon）、再生纤维素、聚偏氟乙烯（PVDF）、聚四氟乙烯（PTFE）、聚丙烯（GHP）和聚醚砜（PES）6种材质的亲水性微孔滤膜，滤膜外壳材质均为聚丙烯。由图3可知，材质为RC时不同目标化合物的平均回收率为77.2%~111%，均能满足检测要求，因此选择RC材质的微孔滤膜进行样品预处理。

**图3:**
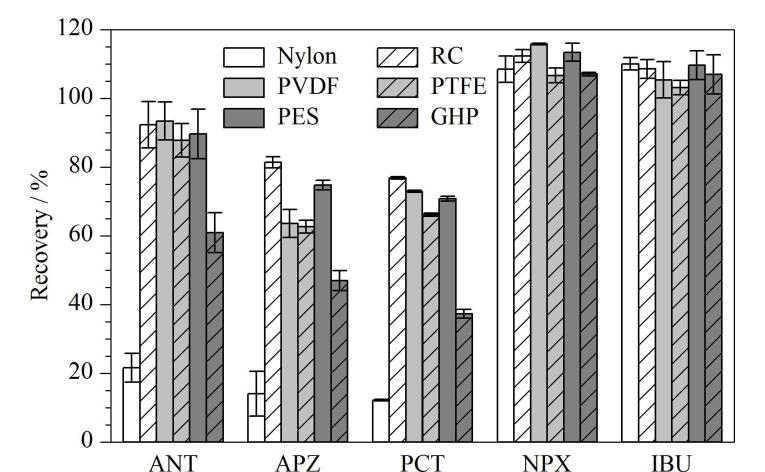
不同滤膜材质对目标化合物回收率的影响 （*n*=3） RC： regenerated cellulose； PVDF： polyvinylidene fluoride； PTFE： polytetrafluoroethylene； PES： polyethersulfone； GHP： polypropylene.

#### 2.3.2 在线萃取条件优化

在线SPE过程与传统的离线SPE过程类似，萃取柱、上样溶剂和淋洗条件等因素对目标化合物的富集净化效果、峰形和方法灵敏度都有影响。实验选取PLRP-S（12.5 mm×2.1 mm，15~20 µm）和Oasis HLB（30 mm×2.1 mm，20 µm）2种SPE柱进行考察，发现使用2种SPE柱萃取的5种目标化合物在峰形和响应值方面差异不明显，均能满足检测需求，综合考虑经济等因素后选择PLRP-S柱进行在线富集。

本方法旨在大体积上样分析环境水样，因此所用的上样溶剂为水。淋洗溶剂用于去除样品中的干扰物质，降低其对目标化合物离子化带来的负面影响。实验发现在纯水中添加0.05%甲酸作为淋洗溶剂，可以明显提高萘普生和布洛芬的回收率，同时增加氨基比林和非那西丁的分离度（见图4），因此选择0.05%甲酸水溶液作为在线固相萃取的淋洗溶剂。实验还对不同的上样体积进行了比较，结果表明上样体积增大，响应值随之提升，但基质效应也更明显，在确保方法灵敏度满足检测要求的情况下，选择上样体积为0.9 mL。

**图4:**
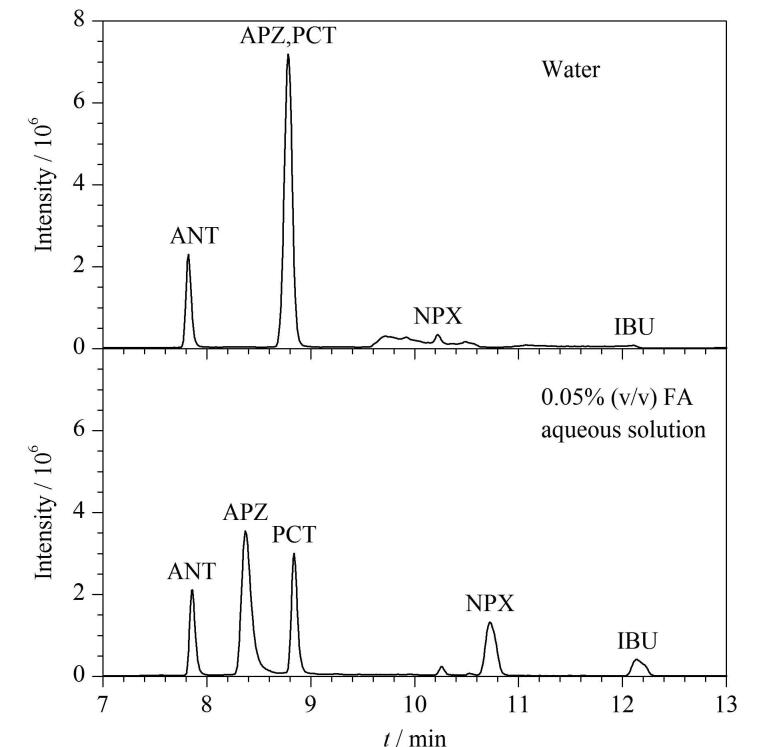
采用不同淋洗溶剂时目标化合物的色谱图 FA： formic acid.

### 2.4 方法学验证

#### 2.4.1 线性关系和检出限

配制5种解热镇痛药物的系列标准工作溶液，按确定的分析条件进行测试。以目标化合物质量浓度与内标质量浓度的比值为横坐标（*x*），对应的目标化合物峰面积与内标峰面积的比值为纵坐标（*y*），绘制工作曲线。根据HJ 168-2020的要求，按MDL=*t*_（_ *_n_*_-1，0.99）_×*S*计算方法检出限，4倍MDL作为方法定量限（MQL）^［18］^。结果表明，目标化合物在线性范围内线性均较好，线性相关系数（*r*）为0.998 5~0.999 6，5种解热镇痛药物的MDL为0.05~0.20 ng/L，MQL为0.20~0.80 ng/L，结果见表3。

**表 3:** 目标化合物的线性范围、回归方程、相关系数、方法检出限及方法定量限

Compound	Linear range/（ng/L）	Regression equation	*r*	MDL/（ng/L）	MQL/（ng/L）
IBU	1.0-100	*y*=1.48*x*+0.32	0.9985	0.20	0.80
ANT	0.5-100	*y*=3.53*x*-0.13	0.9995	0.05	0.20
APZ	0.5-100	*y*=10.89*x*-0.75	0.9996	0.09	0.36
PCT	0.5-100	*y*=4.85*x*-0.17	0.9995	0.11	0.44
NPX	0.5-100	*y*=2.10*x*-0.15	0.9989	0.05	0.20

*y*： ratio of peak area of analyte to internal standard； *x*： ratio of mass concentration of analyte to internal standard.

#### 2.4.2 回收率和精密度

以某阴性地表水样品作为基质，添加目标化合物标准溶液制备2.0、10.0和50.0 ng/L 3个水平的阳性样品，每个水平样品平行试验6次。按建立的方法进行实验，计算其回收率和相对标准偏差（RSD），实验结果见表4。结果显示，3个加标水平下目标化合物的回收率分别在86.0%~103%、69.1%~103%、64.2%~112%范围内，相对标准偏差分别在2.98%~6.58%、2.17%~8.99%、2.06%~4.54%范围内，方法的回收率和精密度符合方法学验证要求。

**表 4:** 目标化合物在3个加标水平下的平均回收率及相对标准偏差（*n*=6）

Compound	Added/（ng/L）	Recovery/%	RSD/%
IBU	2.0	86.0	4.87
	10.0	103	8.99
	50.0	112	2.06
ANT	2.0	93.5	6.58
	10.0	100	5.62
	50.0	89.4	4.07
APZ	2.0	90.1	4.30
	10.0	74.1	3.61
	50.0	70.8	2.77
PCT	2.0	103	2.98
	10.0	69.1	2.17
	50.0	64.2	4.08
NPX	2.0	98.8	5.76
	10.0	89.9	4.21
	50.0	103	4.54

### 2.5 实际样品分析

应用本方法对钱塘江杭州段6个监测点位水样中解热镇痛药物的含量进行检测，如表5所示，5种解热镇痛药物的检出含量为ND~16.3 ng/L，6份水样中共检出4种解热镇痛药物，其中检出频次较高的是安替比林，在5份水样中有检出；氨基比林在6份水样中均低于方法检出限。因此需对地表水中解热镇痛药物的污染予以重视，加强污染状况和污染来源的调查，切实保障水质和生态环境安全。

**表 5:** 实际水样中5种解热镇痛药物的检测结果 (ng/L)

Sample No.	IBU	ANT	APZ	PCT	NPX
1	ND	5.0	ND	ND	ND
2	ND	0.4	ND	ND	1.1
3	ND	1.4	ND	0.5	2.4
4	ND	ND	ND	ND	ND
5	ND	16.3	ND	0.4	1.6
6	2.0	13.7	ND	ND	2.4

ND： not detected.

## 3 结论

本研究建立了在线固相萃取-超高效液相色谱-串联质谱快速测定地表水中5种解热镇痛药物的方法。该方法操作简便，检出限低，灵敏度高，重复性好。与传统的固相萃取方法相比，避免了繁冗的前处理程序和大量的试剂消耗，分析时间短，15 min即可完成一个样品的分析测定，能够满足解热镇痛药物的快速定性定量测定以及大批量环境样品快速监测的需求，具有广阔的应用前景和推广价值。
